# Exposure to 1950-MHz TD-SCDMA Electromagnetic Fields Affects the Apoptosis of Astrocytes via Caspase-3-Dependent Pathway

**DOI:** 10.1371/journal.pone.0042332

**Published:** 2012-08-01

**Authors:** Yu-xiao Liu, Jun-li Tai, Guo-qing Li, Zhi-wen Zhang, Jing-hui Xue, Hong-sheng Liu, Heng Zhu, Ji-de Cheng, Yuan-ling Liu, An-ming Li, Yi Zhang

**Affiliations:** 1 Department of Neurosurgery, The First Affiliated Hospital of PLA General Hospital, Beijing, China; 2 Department of Cell Biology, Institute of Basic Medical Sciences, Beijing, China; 3 China Telecommunication Technology Labs, Beijing, China; 4 Jian Gong Hospital, Beijing, China; University of Navarra, Spain

## Abstract

The usage of mobile phone increases globally. However, there is still a paucity of data about the impact of electromagnetic fields (EMF) on human health. This study investigated whether EMF radiation would alter the biology of glial cells and act as a tumor-promoting agent. We exposed rat astrocytes and C6 glioma cells to 1950-MHz TD-SCDMA for 12, 24 and 48 h respectively, and found that EMF exposure had differential effects on rat astroctyes and C6 glioma cells. A 48 h of exposure damaged the mitochondria and induced significant apoptosis of astrocytes. Moreover, caspase-3, a hallmark of apoptosis, was highlighted in astrocytes after 48 h of EMF exposure, accompanied by a significantly increased expression of *bax* and reduced level of *bcl-2*. The tumorigenicity assays demonstrated that astrocytes did not form tumors in both control and exposure groups. In contrast, the unexposed and exposed C6 glioma cells show no significant differences in both biological feature and tumor formation ability. Therefore, our results implied that exposure to the EMF of 1950-MHz TD-SCDMA may not promote the tumor formation, but continuous exposure damaged the mitochondria of astrocytes and induce apoptosis through a caspase-3-dependent pathway with the involvement of *bax* and *bcl-2*.

## Introduction

During the past decade, mobile phone use has increased almost 100% in many countries in the world, and such increase has raised concerns about the possible risks to human health. Compared to other organs, the brain is exposed to relatively high specific absorption rates (SAR) due to the close proximity of the cell phone device to user’s head. Thus possible effects of cell phone on the central nervous system need to be tested. Several reports showed no association between mobile phone use and brain tumors while others came to the opposite conclusion. For example, Adey et al. and La Regina et al. found that life span and tumorigenicity of rats were not influenced by EMF radiation exposure [1], [2], but epidemiological studies suggested that exposure to EMF may be associated with an increased incidence of brain tumors, especially glioma [3], [4]. In addition, some reports presented evidences of adverse effects caused by EMF radiation on cells including increased apoptosis [5], inhibition of cell proliferation [6], induction of DNA breaks [7], alteration of the gene expression in different cell types [8], and so on. In spite of previous studies, knowledge about the effects of radiofrequency (RF)/microwave (MW) radiation on human health and about the biological responses to RF/MW radiation exposure remains limited.

The present study aimed to investigate the effects of 1950-MHz TD-SCDMA radiation on two different, normal and transformed, types of rat glial cells in culture. The rat astrocytes were firstly purified by shaker oscillation and confirmed by immunofluorescent staining of GFAP. The purified astrocytes and rat transformed astroglial cells (C6 cell line) were then exposed to 1950 MHz TD-SCDMA microwave radiation in a temperature-controlled exposure system at specific absorption rates for 12, 24 and 48 h, respectively. The structural damage to mitochondria of rat normal astrocytes was observed after 48 h of exposure to 1950-MHz TD-SCDMA EMF, accompanied by cell growth inhibition and apoptosis. However, C6 cells appeared to be unaffected by microwave irradiation, and no significant differences could be detected in any of the parameters studied at any time point. To further understand these phenomena, apoptotic genes (*bax* and *bcl-2*) and proteins (caspase-3) were examined in both types of astroglial cells by real-time PCR and Western-blot respectively. More than 12-fold increase of *bax* and 2-fold decrease of *bcl-2* were observed in rat normal astrocytes after 48 h of exposure, accompanied by a significant increase in caspase-3 protein. In contrast, no significant differences were found between the control and experimental C6 cells. Moreover, tumorigenicity assays in nude mice were performed to examine the effect of 1950 MHz TD-SCDMA microwave radiation on glial tumor formation. No visible difference was observed between the control and experimental groups of both cell types. Therefore, the present study showed no evident effects of the microwave radiation used on glial tumor formation, but implied that continuous exposure to 1950-MHz TD-SCDMA EMF might damage the normal astrocytes in culture via a caspase-3-dependent pathway.

## Materials and Methods

### Animals

Postnatal day one (P1) SD rats were used to isolate the astrocytes; BALB/c immunodeficient mice were used in tumorigenicity assay. All the animals were from the Laboratory Animal Center of the Academy of Military Medical Sciences of China (Beijing). All animal experiments were approved by the Animal Care Board of PLA General Hospital.

### Cell Culture

Primary astrocytes were isolated as previously published [9]. Astrocytes were cultured in Dulbecco’s modified Eagle’s medium containing 10% fetal calf serum (HyClone, Logan, UT),100 U/ml penicillin and 100 g/ml streptomycin. Until 80–90% confluent, cells were passaged. Purity of astrocytes was assessed by immunostaining for glial fibrillary acidic protein (GFAP) (B&D Biosciences). C6 glioma cell lines were preserved in Cell Resource Center of Chinese Academy of Medical Sciences(Beijing) and purchased from ATCC. C6 glioma cell lines were cultured in RPMI 1640 medium containing 10%fetal calf serum,100 U/ml penicillin and 100 g/ml streptomycin at 37°C and 5%CO_2_.

### Immuno-fluorescence Staining

Astrocytes were fixed in 4% paraformaldehyde for 20 minutes. Then they were washed with PBS, incubated the cells with GFAP monoclonal antibodies for 1 hour and washed with PBS for 3 times, before acquiring Images using the fluorescence microscope DAPI (5 µg/ml) was added to dishes.

### Flow Cytometric Analysis

Astrocytes were incubated with the FITC-conjugated monoclonal antibodies: anti-human GFAP for 30 minutes at 4°C; then washed with PBS for 3 times, analyzed by a flow cytomteric analysis using the FACSCalibur (Becton-Dickinson, Mountain View, CA, USA).

### Exposure System

The exposure system was provided by China Telecommunication Technology Labs (CTTL). As shown in Fig. S1, the experimental cells (up to four bottles simultaneously) were subjected to the microwave exposure generated from dipole antenna (SPEAG D1900V3-SN1118, Swiss Federal Institute of Technology (ETH), Zurich, Switzerland) fixed under the cell plates. The antennas were connected to a vector signal source plus a power amplifier, which emitted RF EMF at the same frequency as TD-SCDMA mobile phones, i.e., a 1950-MHz carrier frequency, which is the median value of TD-SCDMA mobile phones of Band A (2010–2025 MHz) and Band F (1880–1920 MHz). The power fed to the antenna was 24 dBm (the nominal maximum power). The parameters were qualified by a forward power sensor and meter before the experiment, and monitored by a backward power sensor and meter during the whole exposure experiment to keep the drift within 5%. The SAR, corresponding to the energetic flux absorbed by the tissues, was 5.36 W/kg, according to the antenna calibration certificate issued by Calibration Laboratory of Schmid & Partner Engineering AG. The method used for determine the SAR value was per IEC 62209-1.

The vector signal source (E4438C) and the power meter with couples of power sensors (E4417A and E9327A respectively) were all from the Agilent company. The power amplifier 75A250AM1 was from the Amplifier Research company. As RF exposure was required to be conducted under 37°C, an appropriate heating system was used to keep the temperature of the cell plates steady at 37°C. In each experiment, cells were divided into 4 groups: unexposed and exposed for 12, 24, and 48 h, respectively. At the end of the exposure, the samples were simultaneously removed and processed for morphology and ultramicrostructure observation, cell viability assay, and total RNA, whole cell, as well as nuclear protein extractions. 37.

### Observation of Cell Morphology and Ultrastructure

After exposure, the morphology of cells in different groups was observed under the microscope. For the ultrastructure observation by transmission electron microscope (TEM), cells were fixed for 4 h at 4°C in 5% glutaraldehyde, fully washed for 3 times in 0.1 mol/L phosphate buffered saline (PBS), post-fixed for 2 h at 4°C in 2% osmium tetroxide, dehydrated in a graded series of ethanol, embedded in Epon 812, cut into ultrathin sections (75 nm), and then stained with uranyl acetate and lead citrate. The sections were finally viewed and recorded with a HITACHI H-600 electron microscope at 80 kV (HITACHI, Tokyo, Japan).

### Cell Apoptosis Assays

After exposure, both floating and adherent cells were collected and assessed for apoptosis. The cells of different groups were washed twice with PBS and collected by trypsinization. After centrifugation at 1200 rmp for 5 min at room temperature, the cells were stained with Annexin V and propidium iodide (1 mg/mL). The distribution of cell apoptosis was determined on a FACScort flow cytometer and analyzed by ModFitLT V3.0 software program.

### Cell Proliferation Assay

After exposure, cells of different groups were seeded in 96-well plates at 1×10^4^ cells/well. The cell proliferation was then evaluated using the CCK-8 assay (C0038, Beyotime Inst Biotech, China) according to manufacturer’s instructions. Briefly, the cells were added with 10 µL of CCK8 solution and incubated for additional 3 h. The absorbance at 450 nm wave-length was determined as the indicator of cell proliferation.

### Semi-quantitative RT-PCR and Real-time PCR

The gene expression of cells was examined with semi-quantitative RT-PCR and Real-Time PCR as previously described [10]. All PCR reactions were performed as follows: 95°C for 5 min; 94°C for 40 s; annealing at various temperatures for 40 s, 72°C for 40 s (25 cycles); 72°C for 10 min, 4°C for 5 min. The forward and reverse primers were described in Table 1.

**Table 1:** The primer sequences for RT-PCR.

name	Primer sequence (sense)	Primer sequence (antisense)
*bax*	GTTGCCCTCTTCTACTTTGC	ATGGTCACTGTCTGCCATG
*bcl-2*	GGTCCTCCAGTGGGTATTT	TCCTCCTGAGACTGCCTTAT
*GAPDH*	AAACCCATCACCATCTTCCA	GTGGTTCACACCCATCACAA

### Western Blotting Analysis

Total proteins exxtracts of each group cells were resolved by 10% SDS-PAGE and transferred on PVDF (Millipore) membranes. After blocking, the PVDF membranes were washed 4 times for 15 min with TBST at room temperature and incubated with primary antibody (rabbit anti-capase-3 polyclonal antibody Abcam). Following extensive washing, membranes were incubated with secondary peroxidase-linked goat antirabbit IgG (Santa Cruz) for 1 h. After washing 4 times for 15 min with TBST at room temperature once more, the immunoreactivity was visualized by enhanced chemiluminescence (ECL kit, Pierce Biotechnology), and membranes were exposed to KodakXAR-5 films (Sigma-Aldrich).

**Figure 1:**
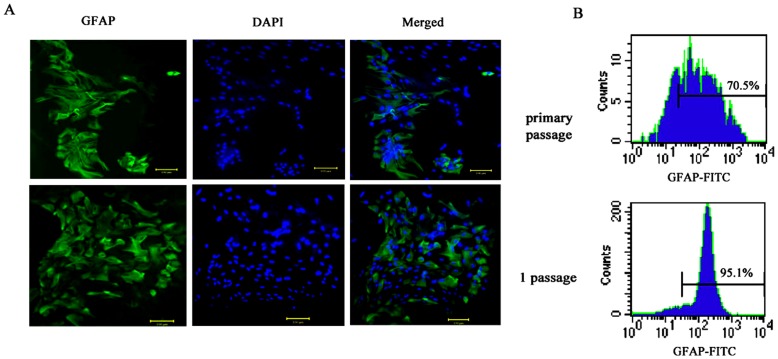
Identification of astrocytes in different passage. Astrocytes in different passage were tested for expression of GFAP (the marker of astrocytes) by immuno-fluorescence staining (A) and flow cytometry (B). The isolated cells display typical astrocyte morphology, bearing satellite processes, and about 70% of the cells initially express GFAP. After subculture, more than 95% percent of derived cells expressed GFAP. Scale bar, 100 um.

### Tumorigenicity Assays in Nude Mice

A total of 1×10^6^ cells suspended in 100 µL 1× PBS were injected into male nude mice subcutaneously. Eight groups of mice were tested and inoculated, respectively with the unexposed, 12 h-, 24 h-, and 48 h-exposed astrocytes, as well as the unexposed, 12 h-, 24 h-, and 48 h-exposed C6 cells. There were 3 mice in each group. Mice were sacrificed at 28 days post-inoculation, and the tumors were excised and the weight of them was measured.

### Statistical Analysis

All the data were expressed as means ± SEM. Statistical significance was determined by using Student’s t-test. Differences were considered significant at P<0.05.

## Results

### Isolation and Identification of the Astrocytes

Astrocytes of higher purity were isolated from the cerebral cortex of P1 rat by dissociation with 0.125% trypsin. The contaminated fibroblasts and other cell types were removed by differential attachment, shaker oscillation, and repeated passages. After purification, the astrocytes of different passages were identified by immunofluorescent staining of the astrocyte-specific cell surface maker–GFAP. The results showed that the isolated cells displayed typical astrocyte morphology, bearing satellite processes, and about 70% of the cells express GFAP initially. After subculture, more than 95% of the cells became positive for GFAP (Fig. 1). The astrocytes of 1–3 passages were used in the subsequent experiments.

### Effect of RF Emission on the Morphology and Ultrastructure of Cells

**Figure 2:**
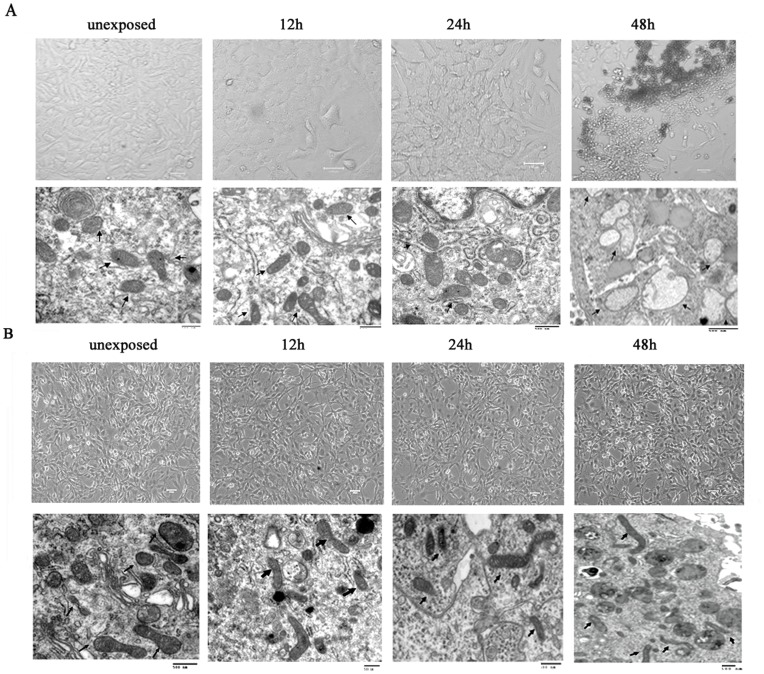
Effect of RF emissions on the morphology and ultra-structure of glial cells. (A) The morphology and ultra-structure of astrocytes were recorded after exposure for 12 h, 24 h, or 48 h. There were no significant differences between control and 12, 24 exposured groups. However, after 48 h of exposure, i.e., part of the astrocytes became shorter and thicker, with some shedding and structural damages in mitochondria as observed under TEM (arrow directed the mitochondria) (B) No significant differences in morphology and ultra-structure of C6 cells were noted among different experimental groups. Scale bar, 100 u m.

In order to elucidate the effect of 1950-MHz TD-SCDMA EMF on the cells of glial origin, normal astrocytes and transformed rat glial cell line–C6 were exposed to 1950 MHz RF field for 12, 24, and 48 h respectively. After exposure, the morphology of the glial cells in different groups was observed by microscopy. The unexposed astrocytes appeared as satellite process-bearing cells in line with previous reports. After exposure for 12 and 24 h, the cell morphology was not significantly different compared with that in control group. However, after 48 h of exposure, many cells became shorter and thicker, and appeared to be with different degrees of degeneration (Fig. 2a). By contrast, the morphology of C6 cells showed no significant differences among groups (Fig. 2b).

Moreover, the normal astrocytes and C6 cells in different groups were collected and fixed. The ultrastructure was recorded by transmission electron microscope. For unexposed astrocytes, clear and wide pericaryon, numerous glial filament, intact mitochondria, rough endoplasmic reticulum, and phagosomes were seen, and there were no significant differences between the control and experimental groups of 12 and 24 h exposure. However, the mitochondria of astrocytes showed uneven distribution, irregular sizes and shapes, swelling, crista break, and cavitation after 48 h of exposure, accompanied by the crescentic margination and fragment of nuclear (Fig. S2) and the morphological change of cells. For C6 cells, the ultrastructure of cells in experimental groups did not change compared to that of control cells. These results indicated that continuous exposure to 1950-MHz TD-SCDMA EMF may cause structural damage, especially the mitochondria damage, in rat normal astrocytes.

### The Growth of Astrocytes was Inhibited after 48 h of Exposure

**Figure 3:**
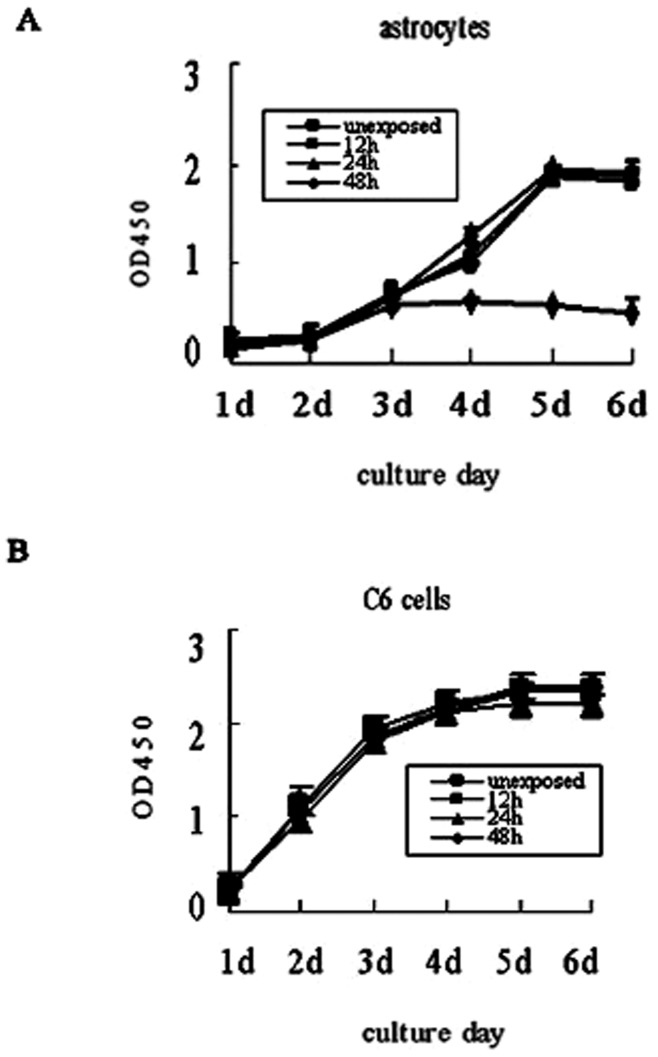
The growth of astrocytes was inhibited after 48 h exposure. (A)The proliferation of astrocytes after exposure (12 h, 24 h, or 48 h) was measured by CKK-8 assay from day 1 to 6. Exposure for 48 h significantly inhibited the growth of astrocyte. (B)No differences were observed in cell growth profile between unexposed and exposed C6 cells. The data represent the mean ± SEM from three experiments.

To investigate the biological effect of T-SCDMA on cell viability, cells of different groups were plated into 96-well plates at 1×10^4^ cells/well after exposure. Cell proliferation was measured by CCK-8 assay from day 1 to 6. As shown in Fig. 3, the viability of astrocytes remained unchanged after 12 and 24 h exposure. However, exposure for 48 h significantly inhibited the growth of astrocytes compared with that of other groups. No differences in the growth of C6 cells were observed in cell growth profile between control and exposed groups.

### The Apoptosis of Astrocytes was Induced after 48 h of Exposure

**Figure 4:**
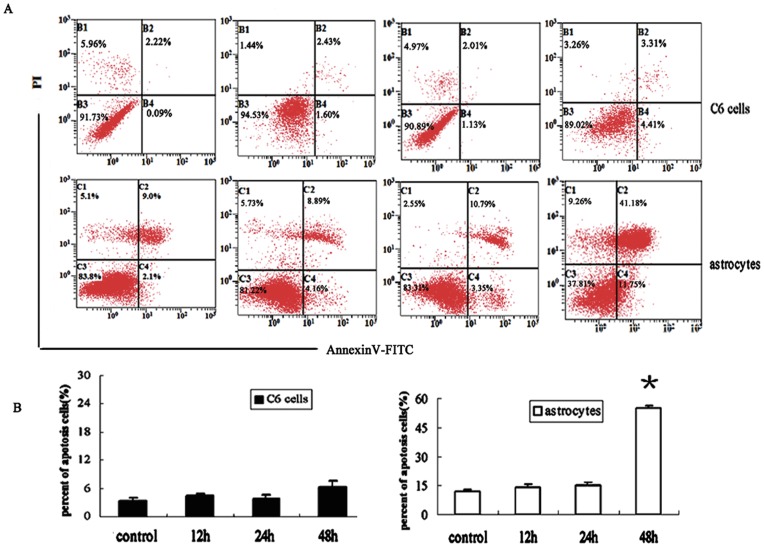
The apoptosis of astrocytes was induced after 48 h exposure. (A) Apoptosis of astrocytes and C6 cells was detected by FITC-conjugated Annexin-V/PI assay after exposure for 12 h, 24 h, or 48 h. Apoptosis of astrocytes was significantly induced by 48 h exposure, and the percent of apoptotic cells increased to about 50%, more than 3 times than that of other groups. (B)Statistical analysis for the percent of apoptosis cells in different groups. The data represent the mean ± SEM from three experiments. *P<0.05 as compared with unexposed groups.

The early and late apoptosis of cells was determined by Annexin V/PI assay (Fig. 4), which measures the transfer of phosphatidylserine from the inner to outer membrane of cells and can detect both early and late apoptotic cells. As shown in Fig. 4, for astrocytes, the apoptosis was significantly induced by 48 h of exposure, and the percent of apoptotic cells increased to about 50%, more than 3 times higher than that of other groups. For C6 cells, no significant differences in early and late apoptosis were observed between the control and exposure groups.

### The Expression of Apoptotic Genes in Glial Cells after EMF Exposure

**Figure 5:**
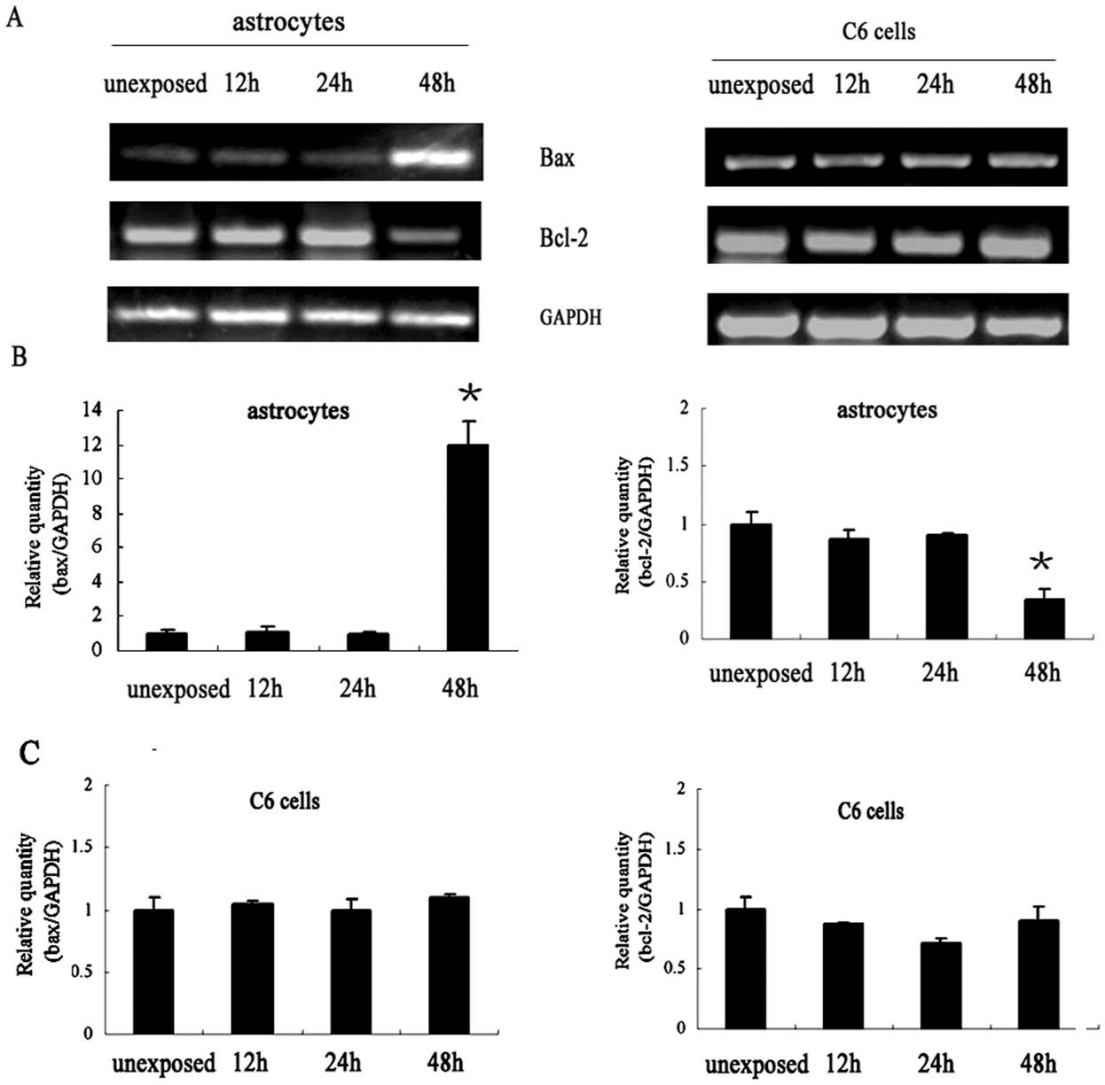
The expression of apoptotic genes in glial cells after EMF exposure. (A) RT-PCR analysis for the expression of *bcl-2, bax* in sham- or RF-exposed cells. A significant down-regulation of *bcl-2* mRNA levels and up-regulation of *bax* mRNA levels were detected in astrocytes after 48 h exposure (B). Real-time PCR analysis for the expression of *bcl-2*, *bax* in sham- or RF-exposed cells. More than 12-fold increase of *bax* and 2-fold decrease of *bcl-2* were detected in 48 h-exposed astrocytes. The average of the normalized ratio of the target gene/GAPDH was calculated. The data represent the mean ± SEM from three experiments. *P<0.05 as compared with unexposed groups.

The apoptotic processes are regulated by several genes including *bax* and *bcl-2*, and both of them play vital roles. Therefore, the possible contribution of *bax* and *bcl-2* in the response to RF radiation in these cells was examined. The total RNAs extracted from either sham- or RF-exposed cells were analyzed by semi-quantitative RT-PCR. As shown in Fig. 5, for astrocytes, a significant down-regulation of *bcl-2* mRNA levels and up-regulation of *bax* mRNA levels were detected after 48 h of exposure, paralleling the cell susceptibility to RF-induced apoptosis. While the expression of *bax* and *bcl-2* was not influenced in 12 h- and 24 h-exposed astrocytes. Real-time PCR analysis confirmed the more than 12-fold increase of *bax* and 2-fold decrease of *bcl-2* in astrocytes after 48 h of exposure. However, for C6 cells, the expression of *bax* and *bcl-2* mRNA did not change due to RF exposure at any time point of exposure. These results suggested the involvement of *bax* and *bcl-2* in RF-induced apoptosis.

### Increase of Caspase-3 was Detected in Astrocytes after 48 h of Exposure

The activation of caspase-3, a member of the cysteine aspartic acid specific protease family, plays a key role in the early phases of apoptosis in mammalian cells. To further study the mechanism of the apoptosis induced by RF radiation, western blot analysis was performed with the whole cell lysates from control cells and cells exposed to RF radiation for 12, 24, and 48 h, respectively.

**Figure 6:**
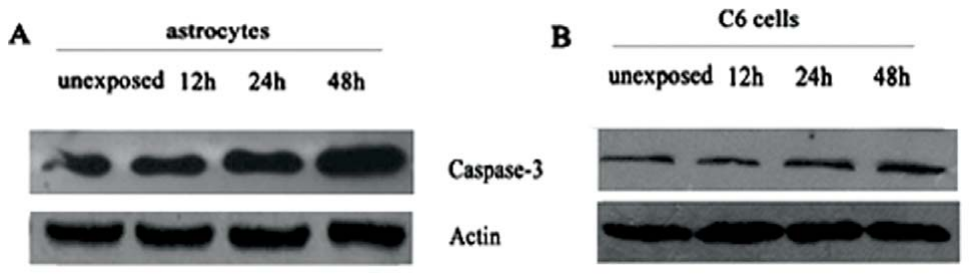
Increased caspase-3 detected in astrocytes after 48 h exposure. (A) Analysis for caspase-3 in astrocytes by western-blot, and a significant increase of caspase-3 protein was detected after 48 h exposure. (B) Caspase-3 expression in C6 cells was not influenced by RF emissions at any exposure time. Using β-actin as the loading control for standardization.

As shown in Fig. 6, in astrocytes, a significant increase of caspase-3 protein was detected after 48 h of exposure, while there were no differences in caspase-3 protein expression between the control and experimental groups after 12 and 24 h of exposure. For C6 cells, the protein expression of caspase-3 was not influenced by RF emissions at any time point of exposure.

### Effect of RF Emissions on the Formation and Development of Glial Tumor

To determine the effect of EMF exposure on the promotion and progression of glial tumors, *in vivo* animal experiments were carried out with unexposed and exposed glial cells respectively. Total of 1×10^6^ cells from control and experimental groups were respectively inoculated into 25 g male nude mice subcutaneously. The tumor formations were observed 4 weeks after the inoculation.

**Figure 7:**
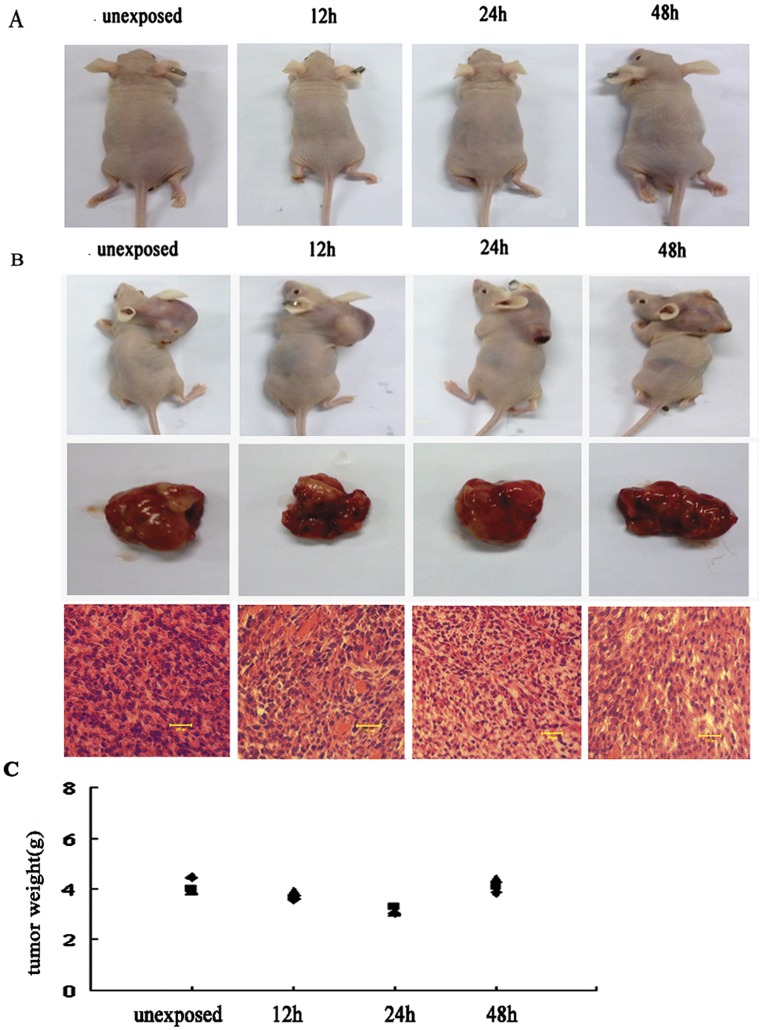
Tumorigenicity assays in nude mice. (A). Tumorigenicity assays for astrocytes. Tumor formation could not be detected either in the unexposed group or in exposed groups (B). Tumorigenicity assays for C6 cells and HE staining of tumors. Tumor could be found in all groups and no visible difference was observed in unexposed and exposed group (C). The weights of C6 tumors in different groups.

The tumor formation by astrocytes was not detected in either control or experimental group from any time point of exposure (Fig. 7A). For C6 cells, although tumor growth was found in all groups during the 4 weeks of follow-up (Fig. 7B), the size and weight of tumors exhibited no significant difference between both control and experimental groups from any time point of exposure (Fig. 7C).

Furthermore, C6 tumor tissues were embedded in paraffin and stained with hematoxylin and eosin (H&E) for histology examination (Fig. 7B), and no visible difference was observed in control and experimental groups. These results indicated that the RF exposure had no significant effect on the generation and development of glial tumor.

## Discussion

Since the expanding use of a global system for mobile communication (TD-SCDMA) equipment in our daily life, RF exposure has become a recent focus as a source of physical stress, especially from a biological and human health perspective. The results of numerous *in vitro* and *in vivo* studies are, however, rather conflicting in terms of the effect of mobile phone on central nervous system and the underlying mechanism. Some researchers found the changes in cell cycle control and apoptosis, gene expression, and tumorigenesis, caused by RF radiations [11]–[13], while others reported no significant effects of RF radiations on cellular systems [14], [15], suggesting that different factors may be responsible for the controversial data obtained so far. Indeed, molecular and cellular reactions in response to extremely low frequency (ELF)-EMF exposure depend on factors such as duration of the exposure, tissue penetration, heat generation, and intensity and frequency of the EMF. In addition, biological responses induced by radiations also depend on the type of the field (static or oscillatory), the waveform (sinusoidal, square, etc), the biological status, and the type of the cells exposed [16].

Currently most papers focus on 900 MHz bands of 2G GSM or CDMA systems [16]–[18], and some on 1800 MHz bands or other systems [19]–[21]. These systems are widely used in the world. Because all kinds of 3G technologies, including WCDMA, CDMA2000, and TD-SCDMA, have been in use for not–so-long, there are only a few relevant reports [22], [23]. Because TD-SCDMA is now widely operated in China, and it covers time-division and code-division technology simultaneously, investigation of the effect of its EMF becomes very significant and important. In this study, the instruments used for EM radiation exposure were all calibrated, professional, and standard equipments, and all the system verification and function monitoring were designed according to the system, to ensure the repeatability and traceability. A 1950 MHz calibrated standard dipole fed with 24 dBm TD-SCDMA signal was used to represent the typical radiation patterns of TD-SCDMA mobile phone under the maximum transmitting power. In the brain, the function of astrocytes is important in the context of biological effects from microwave exposure. This kind of cells is widely distributed in the brain and directly involved in the response to brain damage as well as in the development of brain cancer. Therefore, in this study, the biological responses of rat normal astrocytes and C6 cells were examined after exposure to 1950 GHz TD-SCDMA RF fields.

It is crucial to clearly define the RF exposure period and time point of sampling for evaluating the biological responses to RF radiation. In most of the studies focusing on the cultured cells, the RF exposure period was defined as population-doubling time of the selected cell type, and the period was within 96 h [22]–[26]. Therefore, in this study, the exposure time was set up to 48 h, about 2–4 times of the used cell’s population-doubling time. Another reason for choosing 48 h as the longest exposure time was that the growth of the cells might be interfered by the cell density after 48 h of exposure.

Firstly, we observed the morphological response of two cultured glial cells to RF exposure and we found the differential effect of EMF on both kinds of cells. For C6 cells, no differences in cell morphology and ultrastructure were observed after the exposure to EMF at any time point. In contrast, a significant change of cell morphology and ultrastructure was identified in normal astrocytes after 48 h of exposure, i.e., part of the astrocytes became shorter and thicker, with some shedding and structural damages in mitochondria as observed under TEM. As an energy supply and storage organelle, mitochondria play important roles in cell survival and growth. The change of mitochondrial structure is closely related to energy metabolism of the cells, may influence the cellular function and even cause cell death. The structure and function of mitochondria are complex and sensitive, making it is an important target of many stimulating factors. Our results indicated that mitochondria were the earliest organelle in astrocytes which displayed pathologic changes after continuous exposure to 1950-MHZ TD-SCDMA fields, and we hence speculated that mitochondrial damage might form the basis for changes in the biological features of astrocytes.

*In vitro* study of the apoptosis and proliferation of cells is an important way to assess the influence of RF on cells. If RF fields are capable of influencing tumor promotion, there is a strong possibility that they will correspondingly increase the cell division rate and reduce the cell apoptosis. A number of investigations on the cell proliferation and apoptosis have been conducted, but no general conclusion can be drawn [27]–[32]. The present study has tested the effect of 1950-MHz TD-SCDMA RF field on apoptosis and proliferation responses of two kinds of glial cells, which have been exposed to EMF for 12 to 48 h and assayed for apoptosis and proliferation using Annexin V/PI and CCK-8 assays. Our results indicated that no substantial effects were identified between sham and exposed C6 cells in terms of the growth or apoptosis after exposure to 1950-MHz TD-S-CDMA for 12 to 48 h; on the other hand, a marked decrease in cell proliferation and an increase in cell apoptosis were detected in normal astrocytes after 48 h of exposure to RF, which was paralleled by the morphological changes of cells, implying that 48 h of exposure to 1950-MHz TD-SCDMA RF might exert adverse effect on normal astrocytes but showed no effect on the transformed glial cells.

Furthermore, *in vivo* tumorigenicity experiments indicated that the1950-MHz TD-SCDMA RF field could neither induce tumorigenesis in normal astrocytes nor alter the feature of C6 glioma cells, suggesting that the 1950-MHz TD-SCDMA RF field failed to act as a tumor-promoting agent.

Based on these findings, we further investigated the mechanisms of apoptosis induced by RF exposure. Apoptosis, or programmed cell death, is an important biological phenomenon, because it can provide protection in response to injury to minimize further damages initiated by the injury itself. Although the pathways leading to apoptosis may or may not depend on caspase proteins, caspase-3 is the responsible effector for apoptosis in many situations [33]. In addition, apoptotic processes are regulated by several proteins including bax (an inducer of apoptosis) and bcl-2 (an inhibitor of apoptosis), the critical intracellular checkpoint of apoptosis within a distal common cell death pathway [34], [35]. Thus, to elucidate the pathway involved in RF exposure-induced apoptosis, we analyzed *bcl-2* and *bax* by real-time PCR and caspase-3 by Western-blot. Specifically, we found significant alterations in the transcript level of the two opposing members, *bcl-2* and *bax,* in astrocytes after 48 h of exposure to 1950-MHz TD-SCDMA. The down-regulation of *bcl-2* mRNA levels was accompanied by the up-regulation of *bax* transcript levels after same time of exposure. The protein expression of caspase-3 was also augmented after 48 h of exposure. These results were in accordance with the apoptosis ratio of astrocytes examined by Annexin V/PI assay. In contrast, no significant effect on the gene and protein expression profile was observed in C6 cells after the same treatment. Thus our results demonstrated that the apoptosis of astrocytes induced by RF exposure was mediated by caspase-3-related pathway with the involvement of both *bax* and *bcl-2.*

Therefore, the present study showed that 1950-MHz TD-SCDMA phone emissions exerted differential effects on normal astroglial and transformed glial cells. The variation in culture conditions that might contribute to the observed differences were minimal, because both cells were cultured in an attached manner, and the density of cells was adjusted to ensure the two cell types to be passaged at the same time. Although the culture media for two kinds of cells were slightly different, we anticipated that the media should contribute minimally to this change. To our knowledge, tumor cells showed a stronger ability to proliferate and repair the damages caused by environment. Therefore, it was possible that C6 cells have a stronger inner resistance to EMF exposure.

In summary, this study firstly examined the effects of 1950-MHz TD-SCDMA emissions from a calibrated standard dipole on the biological features of normal or transformed cells of glial origin. Our results confirmed that the 1950-MH TD-SCDMA EMF exhibited no significant effect on cancer promotion, progression, or carcinogenesis of rat astrocytes and C6 cells at the exposure time from 12 to 48 h. However, 48 h of exposure damaged the mitochondria of astrocytes and induced apoptosis through caspase-3-dependent pathway involving *bax* and *bcl-2*. These results are important for properly understanding the mode of action of 1950-MHz TD-SCDMA RF radiation on living organisms, especially in the case of adverse effects. However, further studies are needed to ascertain the effect of EMF exposure on other related cell types and at longer exposure time.

## Supporting Information

Figure S1 **The exposed system.** The Signal generator generates TD-SCDMA modulated signal, and then the signal is output to the Amplifier. The Amplifier amplifies the weak signal to a relative strong level. The Directional Coupler is used to provide additional the monitor path. However, before the directional path is connected to the dipole, its power must be measured by Power Meter PM1 with the Attenuator Att1, which is for protecting the PM1 from damage due to too large power value. At the same time, the reading of PM2 shall be recorded. During the whole experiment, constant reading of PM2 could guarantee the power feed into the Dipole is stable. At last dipole beneath the cell plates radiates electromagnetic exposure.
(TIF)

Figure S2 **Ultra-structure of nucleus in control and exposed-astrocytes.** There were no remarkable changes of the nucleus in astrocytes after exposure for 12 and 24 h. However, crescentic margination and fragment of nuclear was found in 48 h-exposed astrocytes.
(TIF)
