# Supplementary figures and images for: Exposure to 1950-MHz TD-SCDMA Electromagnetic Fields Affects the Apoptosis of Astrocytes via Caspase-3-Dependent Pathway
Source: PLoS One. 2012 Aug 1;7(8):e42332. doi: 10.1371/journal.pone.0042332 (PMC3411641; doi:10.1371/journal.pone.0042332)

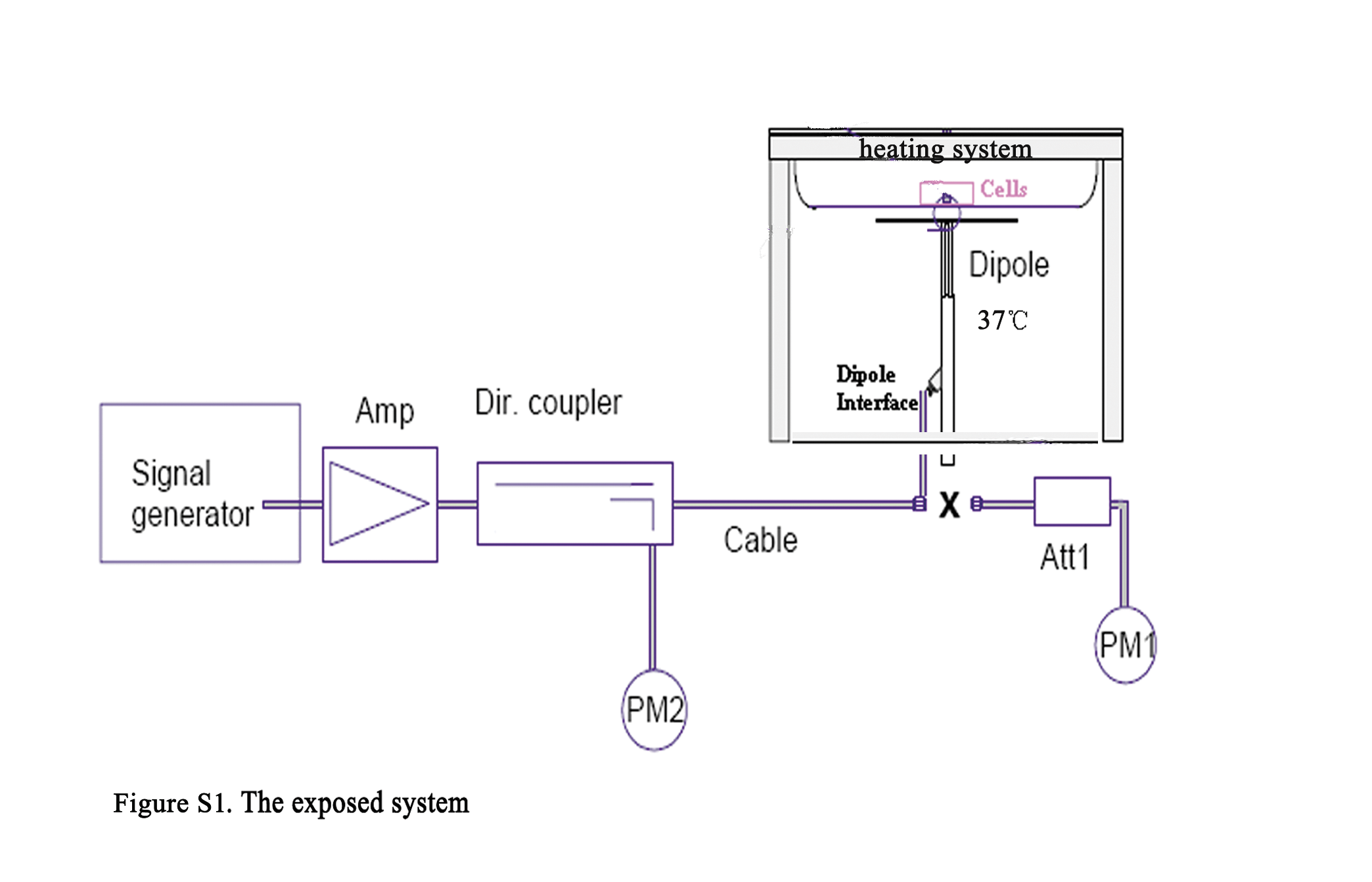

Supplement: Figure S1 — The exposed system. The Signal generator generates TD-SCDMA modulated signal, and then the signal is output to the Amplifier. The Amplifier amplifies the weak signal to a relative strong level. The Directional Coupler is used to provide additional the monitor path. However, before the directional path is connected to the dipole, its power must be measured by Power Meter PM1 with the Attenuator Att1, which is for protecting the PM1 from damage due to too large power value. At the same time, the reading of PM2 shall be recorded. During the whole experiment, constant reading of PM2 could guarantee the power feed into the Dipole is stable. At last dipole beneath the cell plates radiates electromagnetic exposure. (TIF) [file pone.0042332.s001.tif]

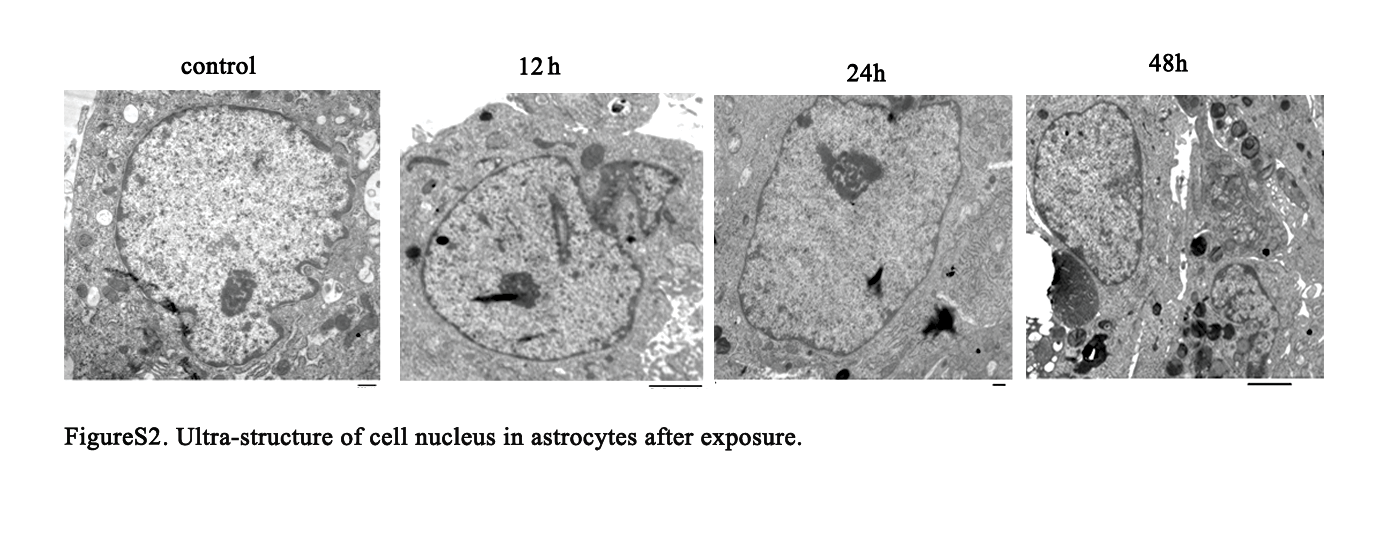

Supplement: Figure S2 — Ultra-structure of nucleus in control and exposed-astrocytes. There were no remarkable changes of the nucleus in astrocytes after exposure for 12 and 24 h. However, crescentic margination and fragment of nuclear was found in 48 h-exposed astrocytes. (TIF) [file pone.0042332.s002.tif]
